# piRNA/PIWI Protein Complex as a Potential Biomarker in Sporadic Amyotrophic Lateral Sclerosis

**DOI:** 10.1007/s12035-021-02686-2

**Published:** 2022-01-11

**Authors:** Rehab F. Abdelhamid, Kotaro Ogawa, Goichi Beck, Kensuke Ikenaka, Eriko Takeuchi, Yoshiaki Yasumizu, Jyunki Jinno, Yasuyoshi Kimura, Kousuke Baba, Yoshitaka Nagai, Yukinori Okada, Yuko Saito, Shigeo Murayama, Hideki Mochizuki, Seiichi Nagano

**Affiliations:** 1 Department of Neurology, Osaka University Graduate School of Medicine, Suita, Osaka Japan; 2 Department of Neurotherapeutics, Osaka University Graduate School of Medicine, Suita, Osaka Japan; 3 Department of Statistical Genetics, Osaka University Graduate School of Medicine, Suita, Osaka Japan; 4 Department of Experimental Immunology, Osaka University Immunology Frontier Research Center, Suita, Osaka Japan; 5 Department of Neurology, Faculty of Medicine, Kindai University, Osakasayama, Osaka Japan; 6 Department of Neuropathology (Brain Bank for Aging Research), Tokyo Metropolitan Geriatric Hospital and Institute of Gerontology, Itabashi, Tokyo, Japan; 7 Brain Bank for Neurodevelopmental, Molecular Research Center for Children’s Mental Development, Neurological and Psychiatric Disorders, Osaka University United Graduate School of Child Development, Suita, Osaka Japan

**Keywords:** Amyotrophic lateral sclerosis, miRNA, piRNA, PIWI protein, TDP-43

## Abstract

The pathological hallmark of the majority of amyotrophic lateral sclerosis (ALS) cases is the mislocalization and aggregation of TAR DNA-binding protein 43 (TDP-43), an RNA-binding protein. Several studies have attributed disease processes of ALS to abnormal RNA metabolism. However, dysregulated biogenesis of RNA, especially non-coding RNA (ncRNA), is poorly understood. To resolve it, RNA-Seq, biochemical, and immunohistochemical analyses were performed on the pyramidal tract of the medulla oblongata of sporadic ALS (sALS) and control postmortem brain samples. Here, we report perturbation of ncRNA biogenesis in PIWI-interacting RNA (piRNA) in several sALS brain samples associated with TDP-43 pathology. In addition, we confirmed the dysregulation of two PIWI homologs, PIWI-like-mediated gene silencing 1 (PIWIL1) and PIWIL4, which bind to piRNAs to regulate their expression. PIWIL1 was mislocalized and co-localized with TDP-43 in motor neurons of sporadic ALS lumbar cords. Our results imply that dysregulation of piRNA, PIWIL1, and PIWIL4 is linked to pathogenesis of ALS. Based on these results, piRNAs and PIWI proteins are potential diagnostic biomarkers and therapeutic targets of ALS.

**Supplementary Information:**

The online version contains supplementary material available at 10.1007/s12035-021-02686-2.

## Introduction

During the last decade, significant progress has been made in identifying genes (~ 50) responsible for familial amyotrophic lateral sclerosis (fALS) [1]. However, fALS constitutes only 10% of all ALS cases. The remaining 90% are termed “sporadic” ALS, and most of these cases have no known genetically inherited component. The lack of an identifiable cause of these sporadic cases makes it difficult to diagnose ALS early enough to distinguish it from other diseases causing similar symptoms.

TAR DNA-binding protein 43 (TDP-43) is a highly conserved and ubiquitously expressed RNA-binding protein, which belongs to the heterogeneous nuclear ribonucleoprotein (hnRNP) family [2]. One of the hallmarks of ALS in the vast majority of both familial and sporadic ALS cases is mislocalization, aggregation, and inclusion formation of TDP-43 protein in brain tissues. The function of TDP-43 strongly suggests its role in RNA metabolism of coding and non-coding RNAs (ncRNAs), including small ncRNAs. Apart from fALS, the only possible way to study the pathogenesis of sporadic ALS (sALS) is to compare patient samples to control samples in order to discover potential gene regulatory targets, which should be beneficial for establishing biomarkers to diagnose the disease and to develop treatment strategies to block their aberrant expression.

**Fig. 1:**
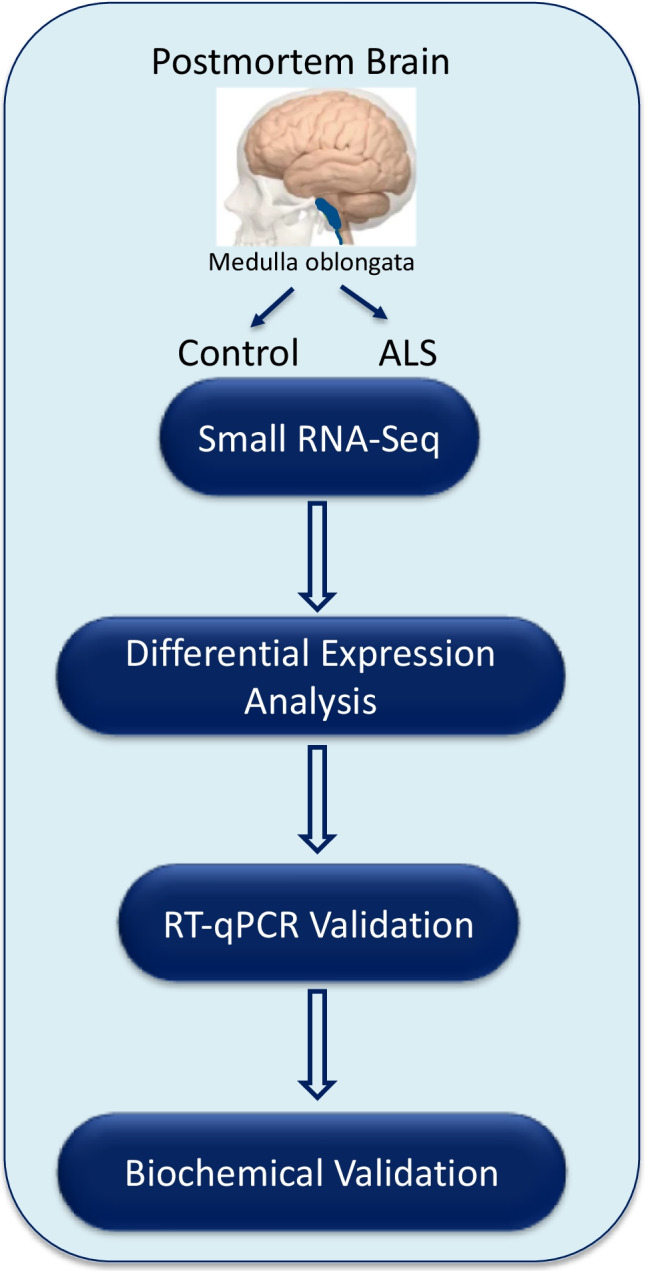
Schematic representation of the experimental design. Postmortem human tissue samples used in this study were collected from ALS cases (*n* = 7) and age- and sex-matched control cases (*n* = 7). RNA-Seq was performed on RNA extracted from the pyramidal tract of the medulla oblongata. Differential expression analysis of small RNAs was performed, followed by validation

Based on these findings, we conducted a pilot RNA-seq study using postmortem brain samples of sALS patients and controls with no known neurological disease (Fig. 1). Differential expression analysis revealed dysregulation in small ncRNAs, particularly in micro RNAs (miRNAs) and PIWI-interacting RNA (piRNA). Owing to recent reports of potential involvement of piRNAs in the etiology of different neurological diseases, such as Rett syndrome, Alzheimer’s disease (AD), Parkinson’s disease (PD) [3], and brain aging [4], we focused on piRNA dysregulation in sALS samples to identify targets of those piRNAs and to determine whether PIWI protein/piRNAs might contribute to ALS pathology.

## Methods

### Postmortem Brain Samples

Postmortem human tissue samples used in this study were collected from the Brain Bank for Aging Research (BBAR) at the Tokyo Metropolitan Geriatric Hospital and Institute of Gerontology (TMGHIG), and the National Center of Neurology and Psychiatry (NCNP) Brain Bank. sALS cases (*n* = 7) were diagnosed based on El Escorial and Airlie House revised criteria with no familial history of the disease and confirmed pathological inclusions of TDP-43 in brain lesions. The stage of TDP-43 pathology that determines the severity of ALS was defined according to the criteria by Brettschneider et al. [5]. Age- and sex-matched control cases (*n* = 7) had no neurological pathologies (Table 1). The pyramidal tract of the medulla oblongata was chosen for this study because this area is rich in axons of upper motor neurons, which are affected in ALS.

**Table 1:**
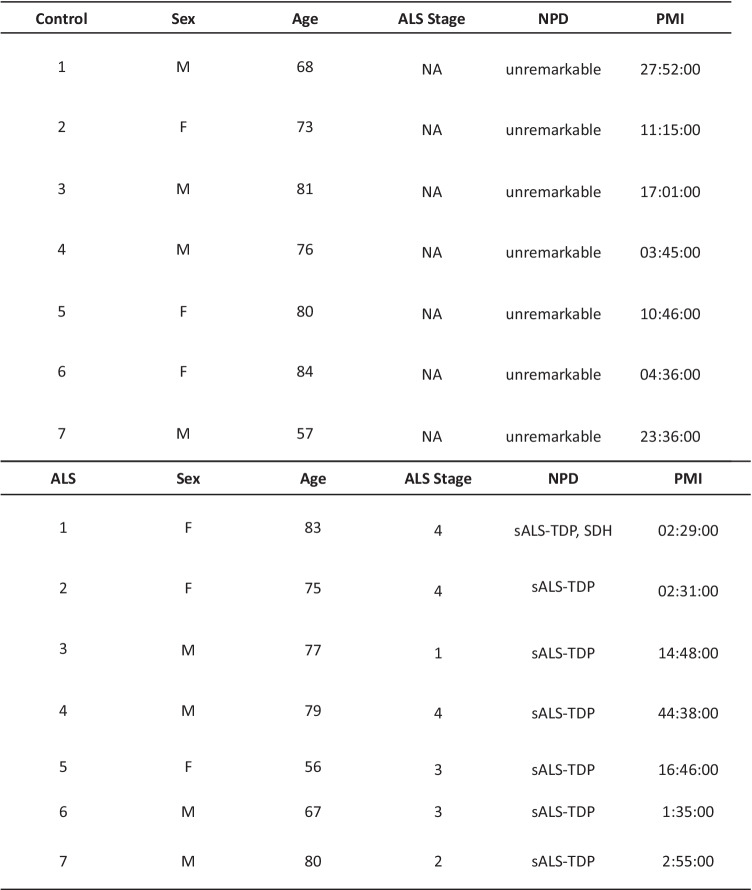
Clinical characteristics of postmortem samples from sALS patients and controls

*ALS* stage was defined according to the criteria by Brettschneider et al. [5], *M* male, *F* female, *NPD* neuropathological diagnosis, *PMI* postmortem interval, *NA* not applicable, *SDH* chronic subdural hematoma, *sALS-TDP* sporadic ALS with TDP-43 pathology

### cDNA Preparation for Small RNA-Seq

Total RNA used for RNA-seq was extracted using Isogen-LS (Nippon Gene # 311–02,501). RNA concentration was measured with a Qubit RNA Assay Kit in a Qubit 2.0 Fluorometer (Life Technologies, CA, USA). RNA integrity was evaluated with an RNA Pico 6000 Assay Kit and a Bioanalyzer 2100 System (Agilent Technologies, CA, USA). The mean RIN for ALS samples was 7.9 (range 6.9–8.5). 50 ng of each RNA sample was used to construct a cDNA library for each sample using a NEXTFLEX Combo-Seq mRNA/miRNA kit (Bio-Scientific), according to the manufacturer’s protocol (Nova-5139–01). cDNAs were sequenced on an Illumina HiSeq 3000 with 74-bp single reads (10 M reads/sample).

### RNA-Seq Bioinformatic Analysis

We excluded adapter sequences using the cutadapt tool V.1.9.2 [6]. Reads shorter than 15 bases were eliminated using trimmomatic version 0.36 [7]. Then, we conducted comprehensive small RNA-seq analysis using the exceRpt extra-cellular RNA processing toolkit, exceRpt version 4.6.3 (https://rkitchen.github.io/exceRpt/) [8].

First, exceRpt automatically excludes low-quality reads. Next, reads aligned to sequences in the Univec Vecscreen database and human ribosomal RNA sequences are removed to filter out contaminants. After filtering, high-quality reads are aligned to the host transcriptome and genome and transcriptome abundances are calculated. In this package, miRbase, circBase, GtRNAdb, piRNABank, and GENCODE are used to assign reads to miRNAs, circular RNAs, tRNAs, piRNAs, and longRNAs.

miRbase (https://mirbase.org/)

circBase (http://circbase.org/)

GtRNAdb (http://gtrnadb.ucsc.edu/)

piRNABank (http://pirnabank.ibab.ac.in/)

GENCODE (https://www.gencodegenes.org/)

### Data Resources

Raw FASTQ files for the RNA-seq libraries have been submitted to the DNA Data Bank of Japan (DDBJ) with Temporary Submission ID: SSUB016807.

### RNA Extraction for Reverse Transcription-Quantitative PCR (RT-qPCR) Validation

Total RNA was extracted from medulla oblongata samples using a mirVana kit (Ambion), according to manufacturer’s instructions. Small RNA (sRNA) was isolated from total RNA using the mirVana kit (Ambion), according to the manufacturer’s instructions for sRNA isolation.

### RT-qPCR for piRNAs

In brief, 3′-ends of 60 ng of sRNA from each sample were ligated to 5′ pre-adenylated 3′-adaptors (5′- rApp/CTGTAGGCACCATCAAT/3ddC-3′) using truncated T4RNA ligase 2 enzyme (NEB). 3′- adaptor-ligated sRNAs were reverse transcribed with ReverTraAce, reverse transcription polymerase (Toyobo) using an oligonucleotide complementary to the 3′-adaptor (IDT) in a MiniAmp plus Thermal Cycler (Applied Biosystems). RT-qPCR was performed using forward primers for each target piRNA and the oligonucleotide complementary to the 3′-adaptor was used as a reverse primer (Table 5). This technique only allows the detection of mature piRNAs. Data were collected in triplicate for each sample on an ABI 7900 Prism qPCR machine and normalized using U6RNA as an internal control. Relative gene-expression levels were calculated using the fold-change method.

### RT-qPCR for Coding Genes

RT-qPCR was performed using ReverTraAce (Toyobo) in a MiniAmp plus Thermal Cycler (Applied Biosystems). The glyceraldehyde-3-phosphate dehydrogenase gene (*GAPDH*) was used as an internal control to normalize coding genes. All quantitative PCR was performed using SYBRGreen qPCR Master Mix (Applied Biosystems) on an ABI 7900 Prism qPCR machine. Primer sets used for RT-qPCR are listed in Table 5.

### Standard Curve Method for RT-qPCR

We used the standard curve method to calculate the average fold change of piRNA expression in ALS samples comparing to control samples.

cDNA generated by reverse transcription for seven controls and seven ALS samples were used for the fold-change calculation. Equal volumes of control cDNA were pooled as a control stock. The control stock was then diluted 10 times with nuclease-free water in a dilution series to produce a control standard curve. sALS cDNA samples were diluted 20 times and then plotted against the control standard curve for each gene in triplicate. *GAPDH* was used as an internal control for normalization.

### △CT Method for RT-qPCR

We used ΔCT method to study piRNA expression of each sample (control and ALS) individually.

The △CT RT-qPCR method was used to calculate the difference in cycle numbers needed to amplify target genes after normalization using the internal control. Therefore, △CT denotes the CT value of a target gene subtracted by the CT value of the internal control. Internal controls for piRNA and coding genes were U6RNA and *GAPDH*, respectively.

### Tissue Lysates and Protein Quantification

CellLytic MT Mammalian Tissue Lysis Reagent (C3228, Sigma-Aldrich), mixed with a protease inhibitor cocktail (1:100, EMD Millipore), was added to frozen tissue in 2-mL Lysing Matrix D tubes (P000912-LYSK0, Precellys) to be homogenized using a MINILYS personal homogenizer (Bretin Instruments). Homogenized tissue lysates were centrifuged and supernatants were used for protein concentration determinations with a Pierce TM BCA protein assay (23225, Thermo Fisher Scientific).

### Immunoblotting for PIWI Proteins

Thirty micrograms of protein from lysates was separated on 5–20% SDS–PAGE gels (E-T520L, ATTO) and transferred to 0.2-μm PVDF membranes (1620177, Bio-Rad). Membranes were blocked for 1 h in EZ blocking solution (AE-1475, ATTO) and then incubated with primary antibodies diluted in Can Get Signal Solution 1 (TOYOBO) overnight at 4 °C. Primary antibodies used included PIWIL1 (1:1000; #701177, Novex), PIWIL2 (1:500; ab181340, Abcam), PIWIL3 (1:500; ab77088, Abcam), PIWIL4 (1:1000; ab111714, Abcam), PIWIL4 (1:1000; PA-49710, Thermo Fisher Scientific), and TXRND1 (1:1000; ab16840, Abcam).

After primary antibody incubation, membranes were washed with 0.1% T-TBS buffer three times (for 15, 10, and 5 min each) before incubation with secondary antibody. Secondary antibodies were HRP anti-mouse antibody (1:10000; NA931V, GE Healthcare) or anti-rabbit antibody (1:20000; NA934V, GE Healthcare) diluted in Can get Signal solution2 (TOYOBO) or EZ block solution and supplemented with STREP-TACTIN (#1616380, Bio-Rad) for marker band detection. After secondary antibody incubation, membranes were washed with 0.1% T-TBS buffer 3 × 10 min before visualization.

### Data Analysis and Statistics (Immunoblotting and RT-qPCR)

Statistics were performed using GraphPad Prism, version9 software. Two-way ANOVA was performed with Bonferroni’s multiple comparison test to compare two or more independent groups. Pairwise comparisons were made using the Mann–Whitney test for △CT with unpaired and nonparametric settings. Pairwise comparisons for fold change using the standard curve method were made using one-sample t-tests (one-tailed Wilcoxon test) with paired and nonparametric settings.

For immunoblot statistical analysis, two-way ANOVA was performed using Bonferroni’s multiple comparison test to compare two or more proteins. Pairwise comparisons were made using the Mann–Whitney test for PIWI proteins with unpaired and nonparametric, two-tailed p-value settings. p-values < 0.05 were considered significant (* indicates *p* < 0.05 and ** *p* < 0.01). All numbers in plots represent means ± SEM.

### Neuropathological Examination

Clinical profiles of patients examined in the present study are shown in Supplementary Table 1. Samples from the lumbar cord (L5) were fixed in 10% buffered formalin. For immunohistochemistry (IHC), 6-µm sections were prepared. Deparaffinized sections were incubated 30 min with 0.3% hydrogen peroxide to quench endogenous peroxidase activity and then washed with PBS. The primary antibody was a mouse monoclonal antibody against PIWIL1 (1:500). Samples were autoclaved 15 min before incubation with antibody. Secondary antibody was goat anti-mouse immunoglobulin conjugated to peroxidase-labeled dextran polymer (Dako Envision + , Dako). Reaction products were visualized with 3,3'-diaminobenzidine tetrahydrochloride (ImmPACT DAB, Vector Laboratories), and hematoxylin was used to counterstain cell nuclei. For double IHC, two primary antibodies were combined, including antibodies against TDP-43 (1:1000, rabbit polyclonal, Proteintech) and PIWIL1 (1:500). Alexa Fluor® 488 goat anti-mouse IgG (H + L) antibody (A-11008, Thermo Fisher Scientific) and Alexa Fluor® 568 goat anti-rabbit IgG (H + L) antibody (A-11004, Thermo Fisher Scientific) were used as secondary antibodies. Sudan Black B treatment was performed to reduce autofluorescence from lipofuscin. Images were obtained using an all-in-one fluorescence microscope (BZ-X710, Keyence).

## Results

### RNA-Seq Analysis for sALS Postmortem Samples

cDNA libraries were prepared using a Combo-Seq kit from seven control and seven sALS postmortem samples (Table 1). This kit was used to analyze poly A-tailed RNA and small ncRNA at the same time. However, it was difficult to get reliable annotation for poly A-tailed RNA during the bioinformatic analysis due to short reads, so we focused on analyzing only reads for small RNA-seq output. Data from two controls (CTR-1 and CTR-7) and sALS (ALS-1 and ALS-3) were excluded from the analysis due to low read numbers obtained from those libraries. Therefore, differential expression analysis was done using five controls and five sALS samples.

**Fig. 2:**
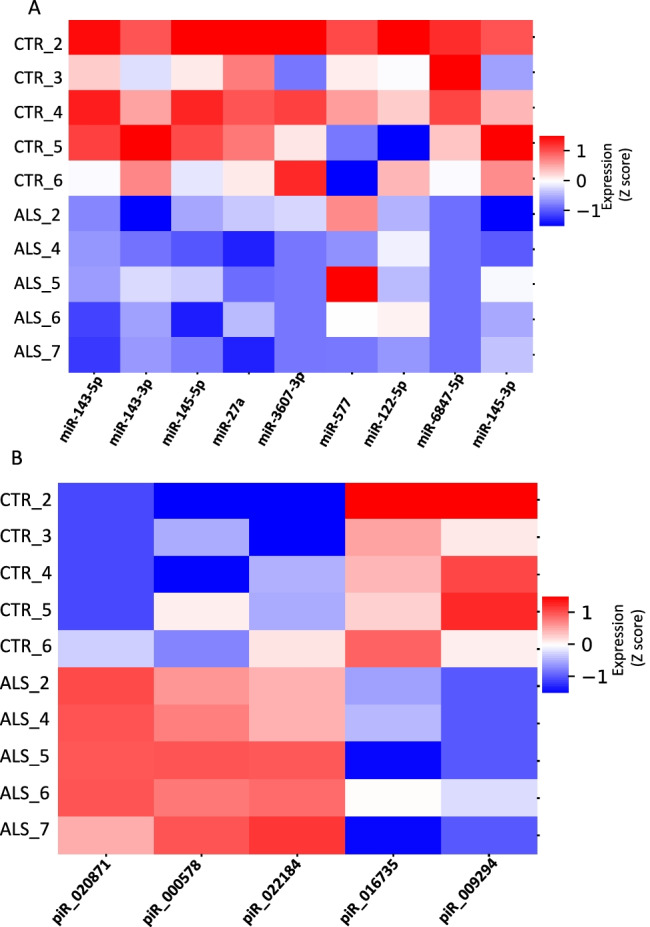
miRNAs and piRNAs are dysregulated in sALS. **A** miRNAs are dysregulated in sALS. Differential expression analysis revealed that nine miRNAs were significantly dysregulated in ALS samples (*p* < 0.05). Data were normalized as the log ^10^ read count per million (RPM). **B** piRNAs are significantly dysregulated in sALS. Three piRNAs were up-regulated in ALS samples (red) and two were down-regulated (blue) (*p* < 0.05). Data were normalized as the log ^10^ read count per million (RPM)

The analysis revealed significant dysregulation (*p* < 0.05) of nine miRNAs in ALS samples compared to controls (Fig. 2A; Table 2). Among dysregulated miRNAs, hsa-miR-143-5p and hsa-miR-143-3p [9–11] were previously reported as dysregulated in cerebrospinal fluid (CSF), serum, and immortalized lymphoblast cell lines (LCLs) derived from ALS patient samples, validating parameters used for data analysis in this study.

**Table 2:**
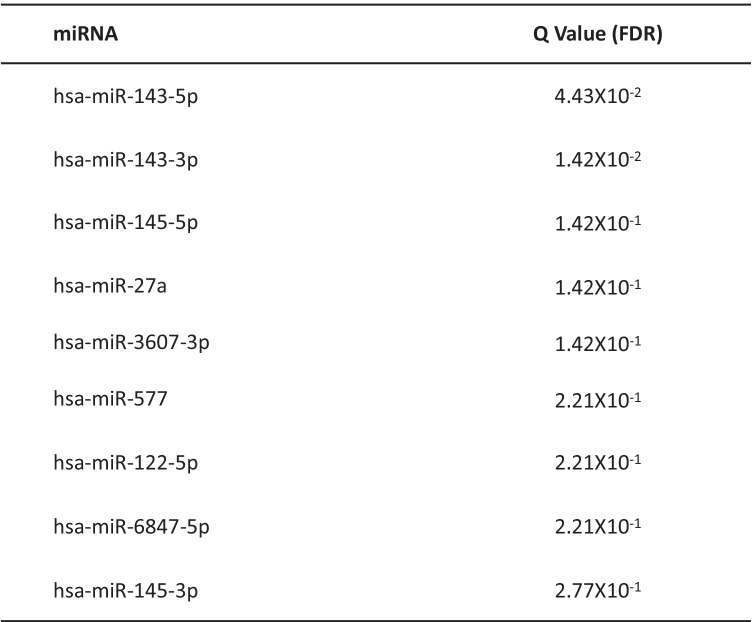
miRNAs that were dysregulated in ALS samples

Two miRNAs, hsa-miR-143-5p and hsa-miR-143-3p, have been previously reported as dysregulated in ALS [9–11]

*FDR* false discovery rate

### piRNA Dysregulation in Postmortem Samples

piRNAs are a class of small ncRNA molecules comprising 24–32 nucleotides, which protect genomic DNA mainly in germ cells by association with PIWI proteins, a subfamily of Argonaute proteins [12, 13]. Five piRNAs were dysregulated in sALS samples (Fig. 2B). Among them, three piRNAs were up-regulated (hsa-piR-000578, hsa-piR-020871, and hsa-piR-022184) and two piRNAs were down-regulated (hsa-piR-009294 and hsa-piR-016735), using a false discovery rate (FDR) < 0.05 and *p *< 0.05 (Table 3).

**Table 3:**
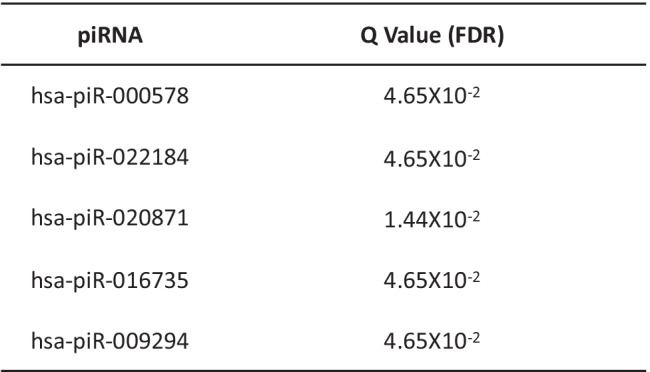
Differentially expressed piRNAs with FDR < 0.05 and P < 0.05

*FDR*, false discovery rate

There are no previous reports of these piRNAs in human brain. The piRNA sequence and target gene sequences were extracted from the PIWI-interacting RNA (piRNA) Database—piRNAdb [14] and predicted target genes for validation from piRBase [15] (Table 4). hsa-piR-33151, which was reported as decreased in serum samples from ALS patients [9], was not detected in our brain samples.

**Table 4:**
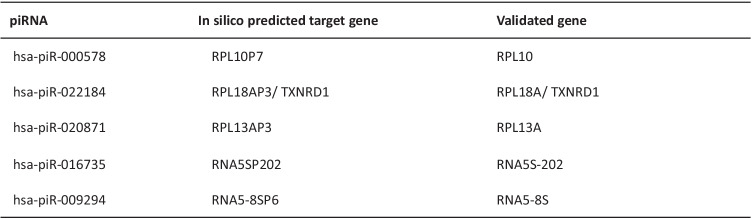
Predicted target genes of dysregulated piRNAs, according to PiRBase and piRNAdb

In order to investigate changes in expression of in silico-predicted target genes for dysregulated piRNAs, we used piRBase and piRNAdb to predict target genes for validation. Most target genes were ribosomal protein pseudogenes, and 5S ribosomal RNA pseudogene transcripts. Due to increased evidence of ribosomal protein dysregulation in ALS, we investigated expression of ribosomal protein coding genes, instead of pseudogenes

**Fig. 3:**
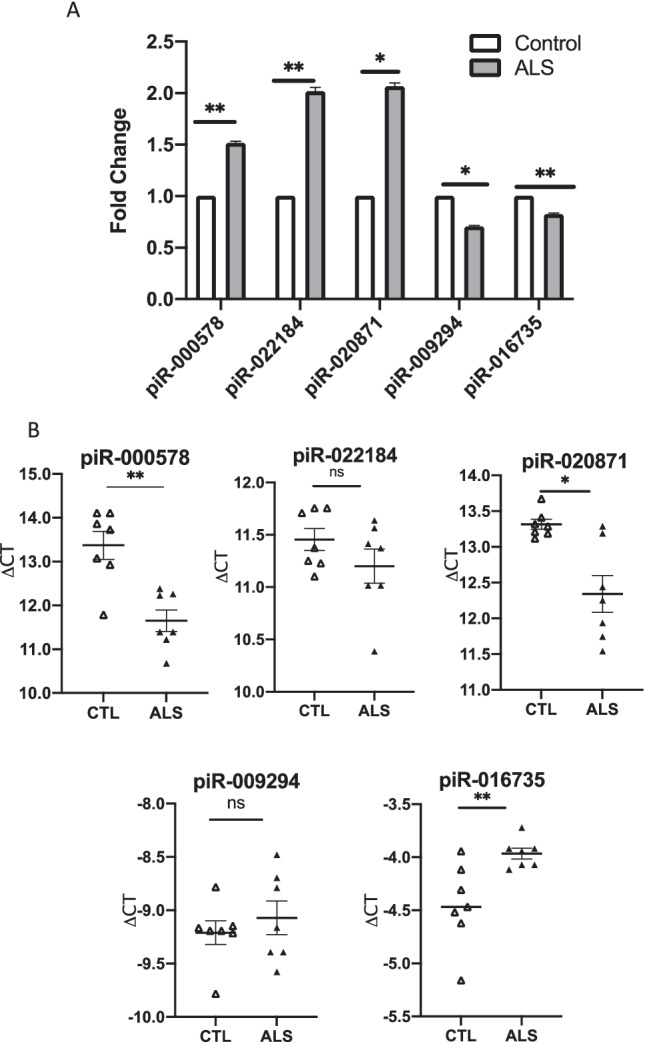
Validation of piRNA expression with RT-qPCR. Dysregulation of five piRNAs was validated using RT-qPCR with two methods, the standard curve method to visualize the overall trend for piRNA perturbation (**A**) and the △CT method to quantify expression in individual samples (**B**). Both methods confirm that these five piRNAs were significantly altered in sALS samples in comparison with controls. **A** Average change in expression of each piRNA in control vs sALS samples. The Wilcoxon signed-rank test analysis was used for statistical calculations. Error bars denote SEMs. **B** piRNA expression by ΔCT in control vs sALS. The Mann–Whitney test was used for statistical calculations. ns: not significant. *p < 0.05, **p < 0.01

Dysregulation of the five piRNAs was validated using RT-qPCR with two methods, the standard curve method to visualize the overall trend for piRNA perturbation and the △CT method to quantify expression in individual samples. RT-qPCR results confirmed the RNA-Seq analysis and these five piRNAs were significantly altered in sALS samples, in comparison with controls (Fig. 3).

### Expression of piRNA Target Genes in Postmortem Samples

**Fig. 4:**
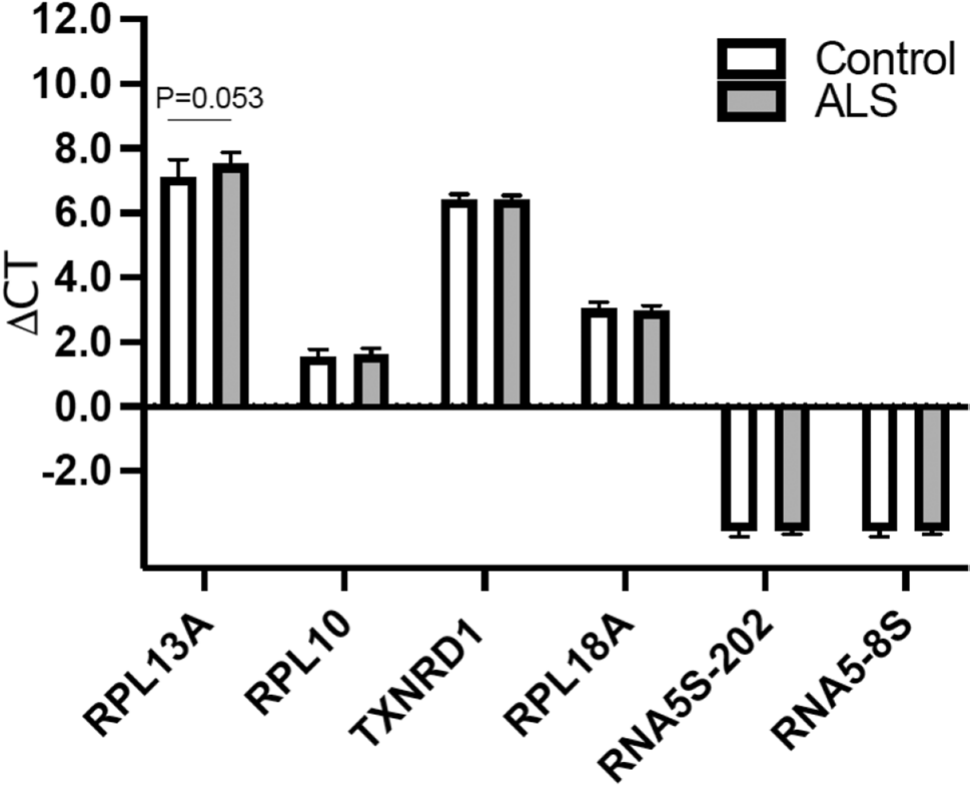
piRNA target genes are mostly ribosomal protein pseudogenes. The △CT method was used to investigate changes in expression of in silico-predicted target genes for dysregulated piRNA, according to piRBase and piRNAdb (Table 4). Except for TXRND1, target genes included ribosomal protein pseudogenes, RPL10P7, RPL13AP3, RPL18AP3, and 5S ribosomal RNA pseudogene transcripts, RNA5SP202 and RNA5-8SP6. We investigated the coding gene expression instead of pseudogenes due to increased evidence of ribosomal protein dysregulation in ALS. There was no notable change in gene expression except for expression of RPL13A (p = 0.053). This suggests that these piRNAs have regulatory effects beyond regulation of in silico-predicted target coding genes

The △CT method was used to investigate the change in expression of in silico predicted potential target genes for dysregulated piRNA, according to piRBase and piRNAdb. This computer prediction method has limitations and may indicate false positive targets. Predicted target genes are listed in Tables 4. Most target genes were ribosomal protein pseudogenes, RPL10P7, RPL13AP3, RPL18AP3, and 5S ribosomal RNA pseudogene transcripts, RNA5SP202 and RNA5-8SP6 except TXNRD1 coding gene. Due to increased evidence of ribosomal protein dysregulation in ALS [16], we investigated expression of ribosomal protein coding genes instead of pseudogenes (Fig. 4). There were no notable changes, except for expression of RPL13A (p = 0.053). We also investigated the downstream translation effect on target genes. For this purpose, we checked the expression level of TXRND1. TXRND1 expression was altered in ALS patients; however, the observation was not statistically significant (Supplementary Fig. 1). These data suggest that these piRNAs have regulatory effects beyond in silico-predicted target coding gene regulation.

**Table 5:**
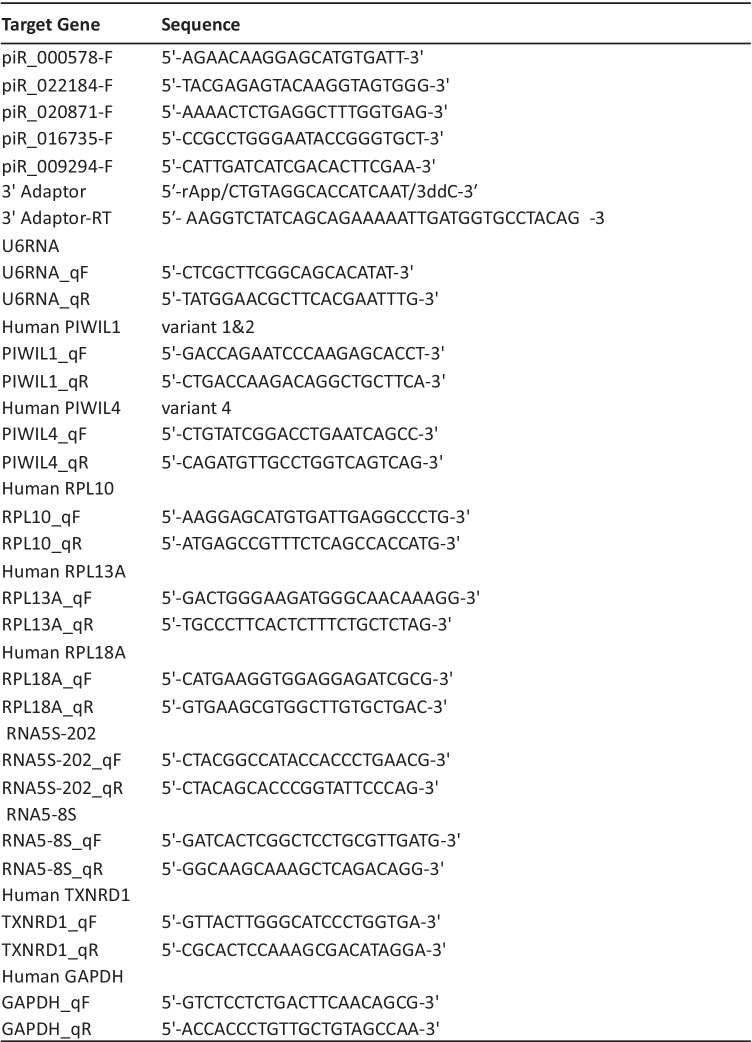
Primers used for qPCR validation of piRNAs and their predicted target genes

### Dysregulation of PIWI Proteins in Postmortem Samples

**Fig. 5:**
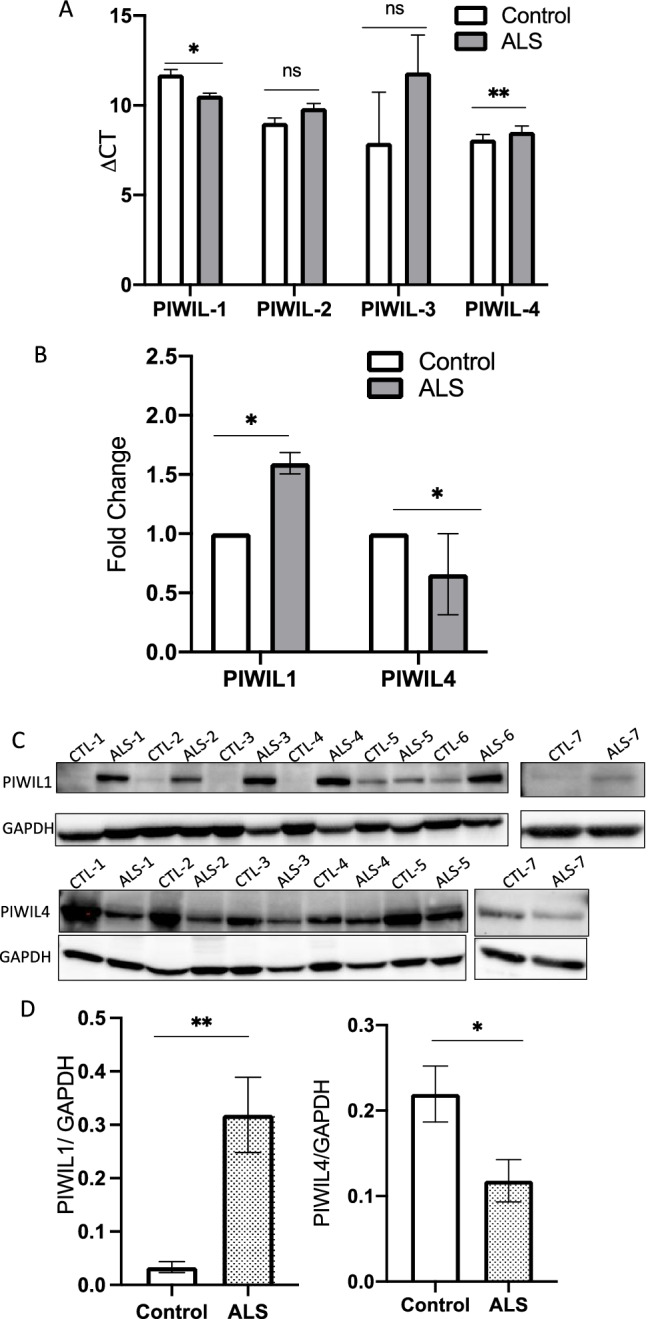
PIWI proteins were dysregulated in postmortem sALS samples. **A** RT-qPCR for PIWIL1, PIWIL2, PIWIL3, PIWIL4 expression of mRNA using the ΔCT method. RT-qPCR using the △CT method for PIWIL1 and PIWIL4 showed significant differences between ALS and control samples, while PIWIL2 and PIWIL3 showed non-significant differences. **B** RT-qPCR results of postmortem PIWIL1 and PIWIL4 expression using a control pool for the standard curve method. One-tailed t-test and the Wilcoxon test were used for statistical calculations. PIWIL1 was upregulated 1.2–1.9-fold in sALS patients compared to the mean of control samples, by the standard curve method. **C** Western blot analysis of PIWIL1 and PIWIL4 in postmortem samples. The increase of PIWIL1 protein and the decrease of PIWIL4 protein were confirmed in ALS samples. **D** Quantitative analysis of PIWIL1 and PIWIL4 in a Western blot normalized to GAPDH. The increase of PIWIL1 protein and the decrease of PIWIL4 protein were statistically confirmed. ns: not significant. *p < 0.05, **p < 0.01

piRNAs are produced by PIWI proteins from different genomic loci and function via the PIWI/piRNA complex. Therefore, we investigated expression differences of PIWI proteins in postmortem samples using RT-qPCR and immunoblotting. PIWIL2 and PIWIL3 were not detected in immunoblots in any of the samples (data not shown). RT-qPCR using the △CT method for PIWIL2 and PIWIL3 showed non-significant differences in △CT between sALS and control samples (Fig. 5A). PIWIL1 was upregulated 1.2–1.9-fold in sALS patients (Fig. 5B) compared to the mean of control samples using the standard curve method. △CT for PIWIL1 was reduced in sALS samples, which means an increase in RNA expression (Fig. 5A). The increase of PIWIL1 protein was confirmed by immunoblot (Fig. 5C, D). PIWIL4 was significantly downregulated an average of 0.6-fold in sALS patients (Fig. 5B) compared to control samples, using the standard curve method. △CT for PIWIL4 was increased in sALS samples, indicating a decrease in RNA expression (Fig. 5A). The decrease of PIWIL4 protein was also confirmed by immunoblot (Fig. 5C, D).

### PIWIL1-TDP-43 Colocalization in Postmortem Samples

**Fig. 6:**
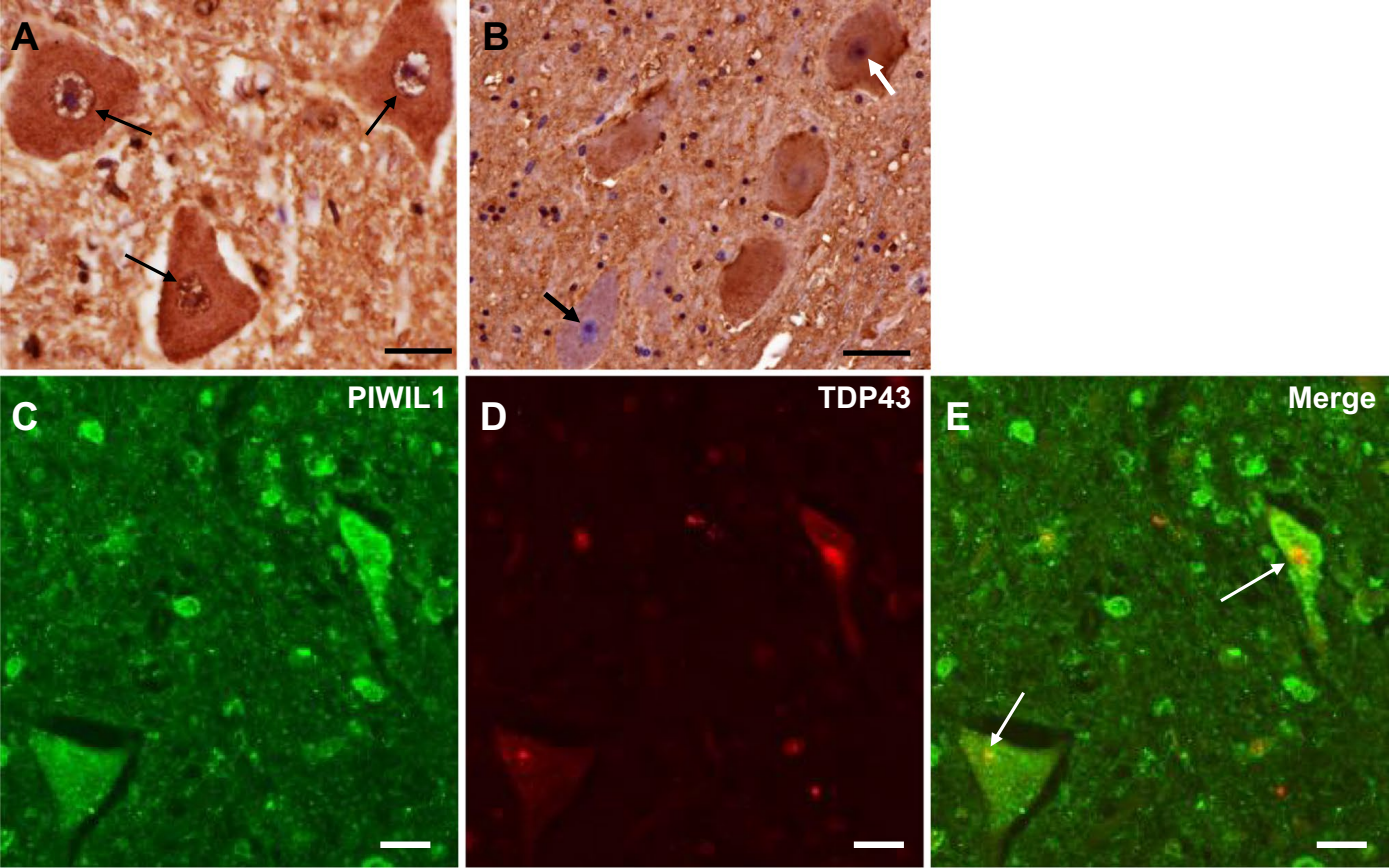
Immunohistochemistry for PIWIL1 in the lumbar spinal cord of control (**A**) and ALS (**B**–**E**) patients. **A** In control patients, cell nuclei, the nuclear membrane (small arrows), and cell bodies of anterior horn cells (AHCs) are immunopositive for PIWIL1. B. In ALS patients, some remaining AHCs have nuclei positive for PIWIL1 (white arrow), while others have PIWIL1-negative nuclei (black arrow). **C**–**E**. In double immunofluorescence staining, PIWIL1 is co-localized with intracytoplasmic inclusions positive for TDP-43 (white arrows) in ALS patients. Scale bar = 20 µm

We examined the expression pattern of PIWIL1 in the lumbar cord (L5) of sALS patients. In control cases, without neurodegenerative diseases, cell nuclei, nuclear membranes, and cytoplasm of anterior horn cells (AHCs) were positive for PIWIL1 (Fig. 6A).

In the lumbar cord from sALS patients, AHCs were reduced in number and 53% of remaining AHCs were atrophic with PIWIL1-negative nuclei (Fig. 6B). In double immunofluorescence staining, PIWIL1 in cell bodies was partially co-localized with TDP-43-positive cytoplasmic inclusions in 30% of AHCs of sALS patients (Fig. 6C–E and Supplementary Table 2).

## Discussion

### piRNA Dysregulation in sALS

We detected and validated dysregulation of five piRNAs in postmortem sALS samples. This is the first evidence of piRNA expression changes in brains of ALS patients, as well as the first report of these piRNAs in human brain. RT-qPCR validation of in silico piRNA target genes did not indicate significant alterations in their expression, suggesting that piRNAs have regulatory effects beyond in silico-predicted target coding-gene regulation. Another possible interpretation of these piRNA alterations is that they are a byproduct or a manifestation of dysregulation in a different genomic region.

Generally, piRNAs contribute to silencing of transposable element (TE) expression, chromatin modifications [17], and mRNA localization [18]. The piRNA pathway targets a subset of LINE1 loci that contain full-length retrotransposon insertions [19], and there is growing evidence suggesting that PIWI proteins and piRNAs function mainly as transposable element regulators in the germline. Brains showing increased expression of retrotransposon elements are likely to be a source of altered piRNA production [20]. On the other hand, TDP-43 was reported to suppress transposable elements in ALS [21], and the loss of nuclear TDP-43 is associated with de-condensation of LINE retrotransposons [19]. The fact that all sporadic ALS samples used in this study manifested TDP-43 proteinopathy suggests that TDP-43 does not protect against TE, which may explain the dysregulation of piRNAs in postmortem medulla samples.

### PIWI Protein Dysregulation as a Potential Cause of ALS

Our results demonstrate dysregulation in PIWIL1 and PIWIL4 in sALS samples. In humans, four PIWI proteins, PIWIL1-4, are involved in piRNA biogenesis and function. Expression of PIWI proteins differs by tissue and organ [22]. PIWIL1 regulates neuronal polarization and radial migration partly by modulating expression of microtubule-associated proteins (MAPs). MAPs are involved in axon extension by directly binding and stabilizing mRNA of target genes, including *MAP1B* [23]. Previous observations show the protective effect of PIWIL1 in neuronal development and microtubule migration. Likewise, a recent study using postmortem samples of brains of Alzheimer’s disease patients showed upregulation of PIWIL1 [24]. Together these lines of evidence suggest that altered expression of PIWIL1 can be explained as a compensatory mechanism for axonal dysfunction. On the other hand, PIWIL1 was previously reported to interact physically with TDP-43 in *Drosophila* [25]. Immunohistochemistry results show PIWIL1 depletion in nuclei of sALS patients compared to controls. Moreover, colocalization of PIWIL1 and TDP-43 was also observed in the cytoplasm of sALS samples, suggesting that increased PIWIL1 and its interaction with TDP-43 in the cytoplasm may contribute to formation of TDP-43 inclusions. This also supports the idea that loss of function in the nucleus or compartmental mislocalization of PIWIL1 in neurons has a drastic impact on neurodegeneration in ALS.

In the current study, PIWIL2 expression was not detected in sALS postmortem samples, supporting the observation by Gasparini et al. [26], that PIWIL2 is expressed in neural stem/progenitor cells, but not in neurons. PIWIL3 also was not detected by immunoblotting in these samples, while PIWIL4 was downregulated by 0.5–0.6-fold in most sALS samples. PIWIL3 has not been reported previously in brain, as it has in ovary, testis, and blood [15]. The role of PIWIL4 in brain is poorly understood, despite recent reports indicating that PIWIL2 and PIWIL4 are associated with autism [27], and that PIWIL4 helps modulate neuronal differentiation from human embryonal carcinoma cells [28]. Disturbance of these tissue specificities of PIWI proteins, or dysfunction of PIWIL4 may also contribute to the disease signature of ALS.

Since PIWIL alterations (PIWL1 and PIWIL4) are observed in the medulla oblongata of ALS samples used in this study, one would expect greater impact on piRNA dysregulation. Technical limitations might explain why only few piRNAs are dysregulated in the same tissue. This could be due to the fact that discovery rate reflects the sequencing depth and the number of useable reads, which may have limited the detection of more target piRNAs. On the other hand, in this study, we have not checked the piRNA population in other areas containing the cell bodies of motor neurons, which might have greater impact.

Overall, our results suggest that dysregulation of the piRNA-PIWI protein axis is associated with pathogenesis of ALS. PIWI protein mislocalization could be an important determinant of TDP-43 accumulation in the cytoplasm. Moreover, dysregulated piRNA and PIWI proteins may be useful as diagnostic biomarkers, as well as gene targets for molecular therapy of ALS.

## Conclusions

This study is the first report of the involvement of PIWI/piRNA metabolism in sALS brain tissues. Our results demonstrate significant dysregulation of five piRNAs, as well as PIWI proteins. They indicate that these piRNAs and PIWI proteins are involved in pathogenesis of ALS, and may be useful as specific disease biomarkers. Furthermore, they may eventually serve as targets for development of novel therapeutics, such as nucleic acid medicines or gene therapeutics.

## Supplementary Information

Below is the link to the electronic supplementary material.

Supplementary file1 (DOCX 2079 KB)

## Data Availability

Datasets generated and analyzed during the current study are available in the DNA Data Bank of Japan (DDBJ) under Temporary Submission ID: SSUB016807.

## Abbreviations

ALS: Amyotrophic lateral sclerosis
fALS: Familial amyotrophic lateral sclerosis
sALS: Sporadic amyotrophic lateral sclerosis
ncRNA: Non-coding RNA
sRNA: Small RNA
miRNA: Micro RNA
piRNA: PIWI-interacting RNA
FDR: False discovery rate
PIWIL1: PIWI-like RNA-mediated gene silencing 1
PIWIL2: PIWI-like RNA-mediated gene silencing 2
PIWIL3: PIWI-like RNA-mediated gene silencing 3
PIWIL4: PIWI-like RNA-mediated gene silencing 4
RT-qPCR: Reverse transcription-quantitative PCR
TDP-43: TAR DNA-binding protein 43
AHC: Anterior horn cell
IF: Immunofluorescence
MAP: Microtubule-associated protein
