# Supplementary material for: piRNA/PIWI Protein Complex as a Potential Biomarker in Sporadic Amyotrophic Lateral Sclerosis
Source: Mol Neurobiol. 2022 Jan 11;59(3):1693–705. doi: 10.1007/s12035-021-02686-2 (PMC8882100; doi:10.1007/s12035-021-02686-2)
Supplement: Supplementary file 1 — Supplementary file1 (DOCX 2079 KB) [file 12035_2021_2686_MOESM1_ESM.docx]

Supplementary Fig. 1

Quantitative analysis of TXRND1 in a Western blot normalized to GAPDH.


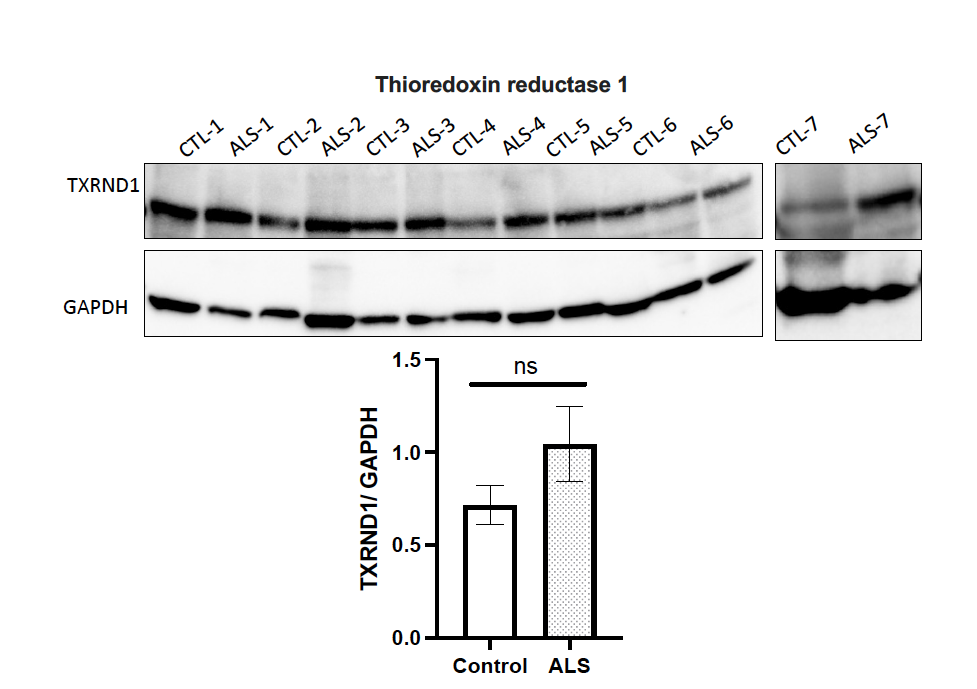


TXRND1 expression had a trend to be altered in postmortem sALS samples, however, this trend is not statistically significant.

ns: not significant

Supplementary Table 1.

Characteristics of postmortem samples for immunohistochemistry and immunofluorescence staining.

M: male, F: female

sALS-TDP: sporadic ALS with TDP-43 pathology

Supplementary Table 2.

Quantitative analysis for PIWIL1 immunohistochemistry and immunofluorescence staining.

IHC: immunohistochemistry

IF: immunofluorescence

AHC: anterior horn cells
